# Supplementary material for: Beyond the game: How social interaction and emotional gratification drive Chinese sports podcast engagement
Source: PLoS One. 2025 Oct 29;20(10):e0335217. doi: 10.1371/journal.pone.0335217 (PMC12571283; doi:10.1371/journal.pone.0335217)
Supplement: S1 Table — presents the alignment between the study’s research questions, methodological approaches, and key findings, showing how cognitive, emotional, and social gratifications were identified and linked to audience engagement on Chinese sports podcasts. (DOCX) [file pone.0335217.s001.docx]

**Supporting Information**

**S1 Table.** Alignment of Research Questions, Methods, and Results

| Research Question | Methodological Approach | Key Results / Findings |
| --- | --- | --- |
| RQ1. What cognitive, emotional, and social gratifications can be identified from user-generated comments on Chinese sports podcasts, and how do these gratifications drive audience engagement? | - Word Frequency Analysis (to identify salient terms) - Co-Word Clustering (to group terms into thematic categories) | - Five primary gratification dimensions emerged: Information Acquisition, Entertainment & Leisure, Social Interaction, Self-Efficacy, and Media Convenience. - Cognitive terms (e.g., “play,” “performance”), emotional terms (e.g., “feel,” “relax”), and social terms (e.g., “family,” “friends”) confirm the tripartite motivational structure. |
| RQ2. How do semantic relationships and thematic clusters in user comments reveal patterns of podcast consumption behaviors on the Himalaya platform? | - Semantic Network Analysis (PMI-based co-occurrence matrices) - Visualization via Gephi | - Semantic clusters highlighted distinct consumption patterns: focus on competitive outcomes (e.g., “win,” “score”), community resonance (e.g., “group,” “friends”), and respect for expertise (e.g., “teacher”). - Network density indicated overlapping cognitive, emotional, and social dynamics in podcast engagement. |
| RQ3. How can the UGT be applied and extended to construct a culturally grounded motivational model of podcast engagement in the Chinese digital media context? | - Mixed-Methods Integration (convergent design combining quantitative text mining and qualitative interpretation) - Theoretical Mapping to the UGT framework | - Extended UGT to account for culturally specific gratifications (e.g., the term “teacher” expressing both authority and respect in Chinese culture). - Constructed a motivational model linking UGT to Chinese digital podcast engagement, showing overlaps between cognitive mastery, emotional resonance, and community identity. |
