# Supplementary material for: Beyond the game: How social interaction and emotional gratification drive Chinese sports podcast engagement
Source: PLoS One. 2025 Oct 29;20(10):e0335217. doi: 10.1371/journal.pone.0335217 (PMC12571283; doi:10.1371/journal.pone.0335217)
Supplement: Attachment — Submitted filename: Rebuttal Letter to Reviewers.docx [file pone.0335217.s003.docx]

**Rebuttal Letter to Reviewers**

Dear Editors and Reviewers,

I am grateful for the opportunity to revise the manuscript titled “Beyond the Game: How Social Interaction and Emotional Gratification Drive Chinese Sports Podcast Engagement.” I deeply appreciate the detailed and thoughtful feedback provided by both reviewers. Below, I provide a point-by-point response detailing how each concern was addressed in the revised manuscript.

| **Reviewer 1 Comments** | **Author Response and Improvements** |
| --- | --- |
| Improve English writing skills, especially in translating professional terms and Chinese contextual vocabulary. For the vocabulary used in the frequency statistics, more detailed explanations and translation explanations should be provided. | I thoroughly revised the manuscript for English clarity and professional tone. Complex terms like “teacher” (老师) were contextually translated, with cultural significance explained (e.g., as a figure of authority rather than simply an educator). Vocabulary from frequency statistics was updated with direct Chinese translations and explanations to aid clarity for international readers. |
| For the conclusion section, please provide a detailed explanation of how the applicability of the theory is proven and provide strong inferences. | The revised conclusion explicitly demonstrates how the Uses and Gratifications Theory (UGT) is supported by findings. The link between self-efficacy and motivational engagement is clearly mapped, and strong theoretical inferences are drawn from empirical data (e.g., identifying podcasts as agentic tools for identity construction). |
| The most important part of the article should be the discussion of Chinese sports Internet social communication, among which the most important part should be the Chinese communication channels, and the similarities and differences with the communication in the same field in other regions (Europe, America, etc.). But these contents are few, I hope the author can add more elaboration on this part. | A new comparative section was added to the Discussion, analyzing Chinese platforms like Himalaya against Western podcast ecosystems (e.g., Spotify, Apple Podcasts). I discussed centralized vs. decentralized media environments and highlighted unique cultural and communicative features in Chinese podcasting. |
| 4. Modern sports have developed for a longer period of time, with more mature communication methods and sports economy itself, but new problems have emerged under modern social media. Issues such as the recent participation of MR.BEAST in the NBA All Star Game, Paul George and Green's blogs, and so on, all reflect the impact of modern social platforms on the dissemination of modern sports. However, this manuscript only mentions word frequency and some basic characteristics, which actually ignores the more subjective influence of modern social media.  But the flaws do not overshadow the merits. The content of this manuscript, the research methods, the actual conclusions, etc., all effectively grasp the direction of the problem, but do not grasp the logical inference of the problem. It should be accepted after revision. | I integrated a subsection on symbolic and subjective media dynamics, noting how influencer participation and multi-platform storytelling shape sports podcast engagement. The example of MrBeast and the rise of athlete-led podcasts was analyzed in light of Chinese equivalents and contrasted with word frequency patterns to add interpretive depth.  The manuscript was restructured to link empirical findings with theoretical claims. Motivational categories (e.g., emotional resonance, media convenience) were tied to behavioral implications, making the logical flow from data to conclusion transparent and academically rigorous |

| **Reviewer 2 Comments** | **Author Response and Improvements** |
| --- | --- |
| Introduction. The intorduction should mention in a clear form the objectives or research questions. At the moment it is not very clear which are the research objectives. | The introduction was rewritten to explicitly include three core research questions, each tied to the UGT framework: cognitive, emotional, and social motivations; patterns in user behavior; and cultural extension of theory. These questions guide the entire manuscript. |
| A comprehensive Literature review related to the researched topic was not included in this article. | To avoid too much word and also based on the journal requirements for research papers, the literature review was included in the introduction. |
| The methodology is not enough explained. The authors mention a mix- of methods, but no research method was mentioned. It is not very clear how data was collected and why that sample was selected. The data analysis process should be explained in more detail and the software used to be mentioned. | The methodology section now details the research design (convergent mixed-methods), sampling (purposive selection of five top channels), data collection (Python scraping tools), and analysis (Jieba segmentation, frequency analysis, semantic networks via Gephi, clustering via Louvain algorithm). Software tools and rationale are clearly stated. |
| The Results section should be organized based on the research objectives or on the analyses mentioned in methodology. Now, the results are put all together. | Results were reorganized into three subsections aligned with research objectives and analytical techniques: motivational themes (from co-word clustering), behavioral signals (from frequency analysis), and conceptual linkages (from semantic network analysis). |
| The Discussions seem to refer to other things than the results. It is not an usual way to make the discussion. Here the authors should debate the results and to compare these ones to other research. | The Discussion was rewritten to follow standard academic practice: (1) restate key findings, (2) interpret each result in context, (3) compare with relevant literature. UGT, Social Identity Theory, and cross-cultural podcast studies were cited to ground the interpretation. |
| In the Conclusion section the contribution of the research should be emphasized. The implications of the research results for theory and practice are not clearly described in the article. A detailed explanations of the author’s recommendations should be included. Research limits should be clearly stated and future research directions should be proposed. In my opinion, the authors have to make efforts to clarify many aspects of the article and report them into a coherent manner. The written in English language should also be improved. | The Conclusion now includes four sub-sections: (1) theoretical contributions, (2) practical implications for content creators and platforms, (3) research limitations (e.g., no demographic segmentation, limited generalizability), and (4) future research directions, including longitudinal and cross-platform studies. |
