# Supplementary material for: Beyond the game: How social interaction and emotional gratification drive Chinese sports podcast engagement
Source: PLoS One. 2025 Oct 29;20(10):e0335217. doi: 10.1371/journal.pone.0335217 (PMC12571283; doi:10.1371/journal.pone.0335217)
Supplement: Attachment — Submitted filename: Reputtal.docx [file pone.0335217.s004.docx]

**Editor’s Comments and Response**

| **Editor’s Comment** | **Response** |
| --- | --- |
| Theoretical and practical implications need to be moved to the Discussion part. | We relocated both theoretical and practical implications into the *Discussion* section, ensuring they are contextualized alongside findings. |
| A clear map of how the mixed methods were used needs to be given. There are different types of mixed-methods research designs; please present which kind of design was adopted and why. | A subsection explicitly stating that the study employed a Convergent Parallel Mixed Methods Design has been added. We provided justification for this choice and included a visual diagram mapping quantitative and qualitative strands to the integration stage. |
| The literature review part is missing. Please add a comprehensive review in this research field. | Literature Review section has been added (before Methodology), structured into three streams: (1) podcast research globally and in China, (2) UGT applications in podcast/media research, and (3) mixed-methods in digital media/audience studies. The section ends with a synthesis that highlights the research gap and positions this study’s contribution. |
